# Insights into the effect of mixed engineered nanoparticles on activated sludge performance

**DOI:** 10.1093/femsec/fiv082

**Published:** 2015-08-04

**Authors:** Samuel Eduok, Callum Hendry, Robert Ferguson, Ben Martin, Raffaella Villa, Bruce Jefferson, Frédéric Coulon

**Affiliations:** School of Energy, Environment and Agrifood, Cranfield University, College Road, Cranfield, Bedfordshire MK43 0AL, UK

**Keywords:** activated sludge, engineered nanoparticles, nano-tolerant microbial species

## Abstract

In this study, the effects, fate and transport of ENPs in wastewater treatment plants (WWTP) were investigated using three parallel pilot WWTPs operated under identical conditions. The WWTPs were spiked with (i) an ENP mixture consisting of silver oxide, titanium dioxide and zinc oxide, and (ii) bulk metal salts. The third plant served as control (unspiked). ENP effects were evaluated for (i) bulk contaminant removal, (ii) activated sludge (AS) process performance, (iii) microbial community structure and dynamics and (iv) microbial inhibition. ENPs showed a strong affinity for biosolids and induced a specific oxygen uptake rate two times higher than the control. The heterotrophic biomass retained its ability to nitrify and degrade organic matter. However, non-recovery of ammonia- and nitrite-oxidizing bacteria such as *Nitrosomonas*, *Nitrobacter* or *Nitrospira* in the ENP spiked reactors suggests selective inhibitory effects. The results further suggest that ENPs and metal salts have antimicrobial properties which can reduce synthesis of extracellular polymeric substances and therefore floc formation. Scanning electron microscopy evidenced selective damage to some microbes, whereas lipid fingerprinting and 454 pyrosequencing indicated a temporal shift in the microbial community structure and diversity. *Acidovorax*, *Rhodoferax, Comamonas* and *Methanosarcina* were identified as nano-tolerant species. Competitive growth advantage of the nano-tolerant species influenced the removal processes and unlike other xenobiotic compounds, ENPs can hasten the natural selection of microbial species in AS.

## Abstract (precis)

The work endeavours to establish linkage between different scales of characterization to establish the true influence of ENP input in wastewater treatment plant.

## Graphical abstract

**Graphical Abstract Figure.**
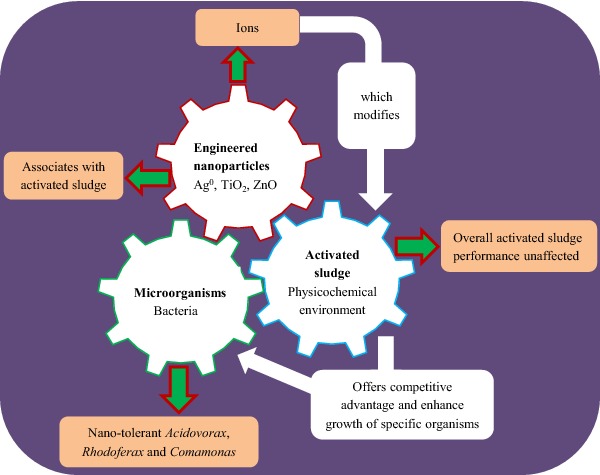
The work endeavours to establish linkage between different scales of characterization to establish the true influence of ENP input in wastewater treatment plant.

## INTRODUCTION

The use of engineered nanoparticles (ENPs) in consumer and industrial products and concerns surrounding their potential effects on the environment and human health cannot be dismissed. There is a compelling need to investigate the effects, fate and transport of ENPs in wastewater treatment plants (WWTPs) which can serve as primary sink and source for aged ENPs and nano-enabled waste. The negative effects of bulk metal oxide salts on wastewater microorganisms as parent material for ENPs suggest that metal oxide nanoparticles can exert similar effects on wastewater organisms (Batley, Kirby and McLaughlin 2012). However, ENP effects on microorganisms in complex environment such as wastewater are not clearly defined and at the moment, information on the long-term effect of mixed ENPs such as Ag^0^, TiO_2_ and ZnO released from consumer products into wastewater or present in activated sludge (AS) is scanty. Moreover, there are uncertainties in relation to ENPs effect on the biologically mediated contaminant removal processes occurring in WWTP (Eduok *et al.* 2013). Although pristine ENPs have antimicrobial effects, information on the effect of aged ENPs on ecologically sensitive organisms in wastewater and the environment is still scarce (Liang, Das and Hu 2010). ENPs in wastewater can lead to reduced efficiency or complete failure of nitrification and subsequent pollution of the environment during effluent discharge. For instance, silver nanoparticle (Ag^0^) can inhibit the removal of nitrogenous material during wastewater treatment (Liang, Das and Hu 2010), and the effect can be magnified in the presence of other ENPs such as titanium dioxide (TiO_2_) and zinc oxide (ZnO). ENPs exert their negative effects via sorption to microbial cell wall, cell wall/membrane disrupted by lipid peroxidation, alteration of cell permeability by producing reactive oxygen species (ROS) and ions (Batley, Kirby and McLaughlin 2012). Apart from the antimicrobial effects, ENPs can enhance growth and increase microbial reaction rates (Hilderbrand, Mackenzie and Kopinke 2009). Notable examples include dechlorination of polychlorinated biphenyl congeners in sediment matrices by *Shewanella oneidensis*-palladium nanoparticle (De Windt *et al.* 2006), batch anaerobic reduction of nitrate by integrated nanoscale zero-valent iron and microorganisms (Shin and Cha 2008), and enhanced dehydrogenase activity of soil microorganisms (Cullen *et al.* 2011).

These pieces of compelling evidences suggest that understanding the mechanism of action and possible use of ENPs as nanocatalyst to augment the metabolic activities of indigenous microbial species involved in bulk contaminant removal during wastewater treatment can have beneficial effects.

To date, laboratory-scale studies on pure culture and limited studies in complex environmental matrices such as the AS that contains diverse microorganisms are available (Liang, Das and Hu 2010; Hou *et al.* 2012; Sun, Sheng and Liu 2013). Extrapolating the negative effect on monocultures to mixed population to explain the probable behaviour of ENPs in AS poses a challenge because the different condition and synergistic activities of microorganisms can influence the outcomes (Eduok *et al.* 2013). Thus, in this study three continuous flow pilot-scale WWTPs were used as realistic environment to assess the effects of ENPs relative to their bulk metal forms on (1) contaminant removal and (2) microbial structure and diversity and activity.

## MATERIALS AND METHODS

### Pilot plant set up

Three parallel pilot-scale plants, each consisting of primary clarifier (180 L), secondary clarifier (∼150 L) and circular aeration tank (∼300 L), fitted with submerged diffusers for aerating and mixing the sludge were used in the study (Fig. S1, Supporting Information). As a start-up material, return activated sludge (RAS) from a full-scale municipal wastewater treatment works (Anglian Water, Cotton Valley, UK) and settled wastewater from Cranfield University domestic wastewater treatment plant (CUDWTP) on a 50:50 ratio was fed into the aeration tanks maintained at 20 ± 5°C. The settled wastewater influent from CUDWTP was fed into the primary clarifiers at 750 mL min^−1^. Inflow of the settled wastewater from the primary clarifier and recirculation of RAS from the secondary clarifier into aeration the tank was done using peristaltic pumps (505U, Watson and Marlow, UK) at 375 mL min^−1^. The plants were operated at a fixed hydraulic retention time (HRT) of 8 hours, sludge retention time (SRT) of 10 days and subsequently operated over 3 SRT (30 days) to stabilize. Identical conditions were maintained in the three plants with exception that treatment lines 1 and 2 were spiked with ENPs and bulk metal salts, respectively, whereas treatment line 3 served as control (unspiked). The three ENPs used were chosen based on their wide application in many consumer products with particle size of 20 nm for Ag^0^ and ZnO and 21 nm for TiO_2_. The ENPs are most likely to be transformed and accumulated in biosolids from wastewater treatment processes and ultimately in the soil. Silver was a proprietary solution of Ag nanoparticles coated with polyvinylpyrrolidone. Zinc was a high-purity and high-quality zinc oxide nanopowder commercially known as Nanosun. Titanium was a high-purity titanium oxide nanopowder commercially known as Aeroxide P25 (Degussa, Germany). The solution of mixed ENPs was made up of 0.01 mg L^−1^ Ag^0^, 0.08 mg L^−1^ TiO_2_ and 0.12 mg L^−1^ ZnO and the AS was spiked at the rate of 0.14 mL min^−1^ (equivalent to 0.67 mL^−1^ L day^−1^) for 315 days (see Fig. S1 and Table S1, Supporting Information). An equivalent concentration of mixed metal salts comprising silver nitrate (AgNO_3_), TiO_2_ and anhydrous zinc nitrate (Zn (NO_3_)_2_.6H_2_O), and unspiked sludge (control) was used for comparison. The mixed ENP and metal salt suspensions were maintained in dispersed state by continuous stirring at 200 rpm.

### Sampling and analysis

Duplicate samples of influent, effluent and mixed liquor suspended solid (MLSS) samples (1 L each) were collected daily for 20 days and subsequently on weekly basis for analysis. Physicochemical analysis of the MLSS, influent and effluent was determined within 2 hours of sampling by measurement of the pH, suspended solids (SS), total volatile solids, and sludge volume index (SVI) according to standard methods (APHA 2005). The influent and effluent NH_3_^−^-N, NO_2_^−^-N, NO_3_^−^-N, total nitrogen and COD were measured by Hach's vial methods (Camlab and Merck) adapted from standard analytical method (APHA 2005). Particle/floc size of biomass was measured using Malvern mastersizer 2000 (Malvern Instrument, Worcestershire, UK). Treatment efficiency (%) was calculated using the formula: (Influent –Effluent) (100) / Influent. It is worth mentioning that the pilot plants were not designed to remove phosphate, sulphate or denitrify because of the alternating aerobic (famine) and anaerobic (feast) conditions required

### Measurement of ENPs and metal salts ionic forms

ENPs and metal salts (Ag^+^, Ti^4+^, Zn^2+^) residual concentration in AS and effluent at various times was measured by Inductively Coupled Plasma Atomic Emission Spectrometer (ICP-AES, Perkin Elmer 4300DV). Briefly, samples were digested prior to analysis using high-purity nitric acid, hydrogen peroxide and hydrofluoric acid in sealed Teflon vessels with microwave-assisted heating. After digestion, demineralized water having a resistivity of 18.2 MΩ cm was used in making the samples into known volume. Concentrations of Ag^+^, Ti^4+^ and Zn^2+^ were measured by ICP-AES calibrated using certified standards. An amount of 5.0 mg L^−1^ Ag, Ti and Zn was prepared from an alternative source stock from that used to prepare the instrument calibration standard and measured with the samples as a quality control. Procedural blanks spiked with equivalent of 4.0 mg L^−1^ Ag^+^, Ti^4+^ and Zn^2+^ were taken through the same procedure as a further quality control measure. ENPs concentrations are reported as Ag^+^, Ti^4+^ and Zn^2+^ because it was difficult to distinguish the nano and bulk forms of the metal oxides in sludge samples. The concentration of residual ENPs and metal salts recovered were corrected against the control using the formula: % recovery = C_spiked sample_ − C_unspiked sample_ / C_added_ × 100.

### Determination of cultivable bacterial growth kinetics

The effect of ENPs and metal salts on the indigenous bacterial population was assessed using a modified plate and colony counting method as described by Coulon *et al.* (2010). Samples were incubated at 35 ± 0.2°C for 24 h and colony-forming units (CFUs) enumerated. Percentage survival (% S) of cultivable bacterial density was determined using the log-transformed CFU mL^−1^ values and the formula: % S = CFU_exposed_ / CFU_control_ × 100 (Diao and Yao 2009). Bacterial growth was estimated spectrophotometrically from the optical density of the broth (Das *et al.* 2011). An amount of 1.0 mL of effluent sample from each treatment was inoculated onto 100 mL of Nutrient broth No. 2 (Oxoid) and incubated at 35 ± 0.2°C and constantly agitated at 170 rpm. At intervals of 1 h, 3 mL of culture was withdrawn from each flask and optical density (OD) of two replicate inoculations measured at 600 nm for 8 h was used to determine effect on bacterial growth.

### Measurement of oxygen uptake rate (OUR)

The OUR of the microbial community was measured according to the Organization for Economic Cooperation and Development guidelines for the manometric respirometry test (OECD 301F; O'Malley 2006). Specific oxygen uptake rate (SOUR) was calculated from the OUR and mixed liquor volatile suspended solids (MLVSS) using the formula: SOUR = OUR × 1000 / MLVSS.

### Electron microscopy

Structural changes on bacterial cells of the floc samples were screened using scanning electron microscopy (SEM). A drop of unwashed AS was placed on silicon square held onto aluminium stub by double-sided carbon tape and allowed to dry without alteration in the floc morphology. The dried sample was sputter-coated with gold/palladium for 1 min to give a thin layer of about 2–3 nm, and examined using a scanning field emission gun electron microscope (FEI XL30 S-FEG).

### Phospholipid fatty acid (PLFA) analysis

After changes in bacterial community structure, PLFA extraction was performed using a modified method of Bligh and Dyer as described by Frostegard, Tunlid and Baath (1993). Briefly, 5 g of freeze-dried sludge samples were extracted using 0.8:1:2 (v/v/v) citrate buffer-chloroform-methanol, subjected to solid-phase fractionation followed by transesterification by mild alkaline methanolysis (Dowling, Widdel and White 1986) to obtain the fatty acid methyl esters (FAMEs). An amount of 200 μL Nonadecanoic acid methyl ester (Sigma-Aldrich, UK) was added as internal standard to each sample after solid-phase extraction. The dried FAMEs were resuspended in 0.2 mL of hexane and analysed by gas chromatography (GC) (Agilent Technologies 6890N) coupled to a flame ionization detector as described by Pankhurst *et al.* (2012).

### 454-Pyrosequencing of AS microbial community

DNA was extracted from 200 mg duplicate AS samples using a MoBio Power Soil kit (MO BIO Laboratories, Inc., UK) and DNA quality checked on 0.8% agarose gel. For amplification of bacterial 16S rRNA gene fragments, PCR primers were adapted for 454 amplicon sequencing by attaching the M13 adapter (**CACGACGTTGTAAAACGA**) to the primer M13–16S-IA-FL (5^′^–**CACGACGTTGTAAAACGA**CCATGCTGCCTCCCGTAGGAGT–3^′^), whereas the 25-mer Lib-L-specific sequence adapter B (CCTATCCCCTGTGTGCCTTGGCAGTC) was followed by the reverse template-specific primer sequence 16S-IA-RL (5^′^–**CCTATCCCCTGTGTGCCTTGGCAGTC**TCAGAGAGTTTGATCCTG-GCTCAG–3^′^). To aid multiplexing different samples, different barcodes were included in the M13 adapter using the 454 sequence adapter A (CCATCTCATCCCTGCGTGTCTCCGAC) and a 454 amplicon sequencing-specific 4-mer amplification key (*TCAG*) followed by a 10-mer barcode sequence (NNNN) (5^′^–CCATCTCATCCCTGCGTGTCTCCGAC*TCAG*NNNN***CACGACGTTGT-AAAACGAC***–3^′^). Each 20 μL PCR mixture contained primers at 10 μM, 10 mM deoxynucleoside triphosphates and 0.2 μL of high-fidelity polymerase (Phusion, Biolabs, New England, UK), 4 μL Phusion 5x buffer (Phusion, Biolabs, New England, UK) and 1.4 μL MgCl_2_. Amplifications were performed using a Biorad C1000 Thermal cycler (BioRad) as follows: 95°C for 5 min, followed by 35 cycles of denaturation at 98°C for 20 s, annealing at 57°C for 20 s and elongation at 72°C for 30 s. Cycling was completed by a final elongation at 72°C for 10 min. Next generation sequencing of all amplicons was completed using the GS FLX System (Roche). Emulsion PCR was carried out according to the manufacturer's instructions (Roche). Samples were multiplexed on a one-eighth section of the pyrosequencing plate. Sequencing resulted in a total of 17 022 bacterial sequences with an average sequence of 1067 in control, 590 in metal salts spiked and 775 in ENP spiked sludge. The obtained sequence data were processed using the Galaxy platform (http://galaxyproject.org/). Sequences analysed were a minimum of 500 bp (mean length average 560 bp). Splitting of sequences into respective samples was carried out using respective barcodes.

### Bioinformatics

The obtained sequence data were processed using the CloVR-16S 1.0 pipeline (http://clovr.org/) (White *et al.* 2011). Briefly, poor-quality sequences were removed using the Qiime script ‘plit_libraries.py’ (http://qiime.org) using the following parameters (minimum sequence length 100 bp, maximum sequence length 2000 bp, maximum homopolymer length 8, minimum quality score 25 and maximum ambiguous bases 0). The Mothur script ‘unique.seqs’ was used to cluster unique sequences and a set of representative sequences was determined. Representative sequences were then searched against the ‘16S rRNA gold database’ to identify putative chimeras using the default parameters. The chimeric sequences were then excluded from further analysis. Sequences were then clustered, aligned and classified using Qiime workflow ‘pick_otus_throigh_otu_table.py’. Sequences were clustered into operational taxonomic units (OTUs) with a 97% nucleotide sequence identity threshold for all reads within an OTU using the Qiime script ‘pick_otus.py’. Representative sequences for each cluster were selected with ‘pick_otus.py’ and classified using the Ribosomal Database Project Bayesian classifier (http://rdp.cme.msu.edu/) at phylum, class, order and family with a confidence threshold of 0.5, with the script ‘assign_taxonomy.py’. Results presented are the number of sequences assigned to OTUs identified at the respective taxonomic levels.

### Statistical analysis

PLFA and 454 pyrosequencing data were log transformed to reduce skewness in distribution, subjected to species-dependent hierarchical cluster analysis and non-metric multidimensional scaling ordination based on Bray–Curtis similarities using PRIMER software (Clarke and Warwick 2001).

## RESULTS AND DISCUSSIONS

### Effects of biosolids on ENPs

Overall, the sorption of ENPs to biosolids was at least two times higher compared to the metal salts which is consistent with the tendency of ENPs to associate with natural organic matter (NOM) (Table 1). For example, Ag^+^ concentration in the biosolids was 32 ± 7 mg kg MLSS^−1^ for the metal spiked sludge compared to 100 ± 30 mg kg MLSS^−1^ in the case of the ENP (Table 1). Similarly, the concentration of Ti^4+^ in the ENP spiked sludge was 2.6 times higher than in control and 1.3 times higher than in metal salt spiked AS. This finding agrees with previous works which showed that ENPs have high affinity for NOM (Kiser *et al.* 2010; Chauque *et al.* 2014). Similarly the respective ions of the ENPs were undetected in the effluent. In contrast, episodic foaming in the metal salt spiked reactor resulted in 1.6 times higher level of SS, and Ag^+^ and Zn^2+^ were detected in the effluent (Table 1). Concentrations in the control waste activated sludge (WAS) samples are indicative of the background concentration of ENPs or metal salts ions in biosolids. Concentration of Ag^+^ in the WAS control (10 mg kg MLSS^−1^) was higher than concentrations previously reported ranging between 2 and 18 μg L^−1^ (Blaser *et al.* 2008) indicating a potential increase in the use of products containing Ag^0^ and discharge of Ag^0^-enriched waste into the CUDWTP.

**Table 1.** Physicochemical characteristics of WAS and influent wastewater.

	Treatments			
Parameter	Control WAS	Metal spiked WAS	ENP spiked WAS	Influent
Ag^+^ (mg L^−1^)*	n.d.	33.3 ± 0.4	n.d.	n.a.
Ti^4+^ (mg L^−1^)*	n.d.	n.d.	n.d.	n.a.
Zn^2+^ (mg L^−1^)*	n.d.	500 ± 400	n.d.	n.a.
Ag^+^ (mg kg MLSS^−1^)	10 ± 0.0	32 ± 1.7	100 ± 30	n.a.
Ti^4+^ (mg kg MLSS^−1^)	1400 ± 372	1810 ± 597	3678 ± 766	n.a.
Zn^2+^ (mg kg MLSS^−1^)	244 ± 17	656 ± 283	1022 ± 134	n.a.
Conductivity (μS)	730 ± 65	761 ± 48	740 ± 66	929 ± 53
Effluent pH	6.5 ± 0.2	6.45 ± 0.3	6.69 ± 0.4	8.06 ± 0.1
NH_3_^−^ (mg L^−1^)	0.2 ± 0.1	0.8 ± 1.6	0.17 ± 0.9	37 ± 6
SVI (mL g^−1^)	85 ± 27	152 ± 68	100 ± 28	na
TSS (mg L^−1^)*	32 ± 11	51 ± 17	33 ± 12	na

Values are mean ± standard deviation of ENPs and metal salts partitioned into WAS from 315 days of treatment. * = concentration in effluent, n.d. = not detected; n.a. = not applicable.

### Effects of ENPs on biological removal of nitrogenous compounds, organic carbon and SOUR

**Figure 1.**
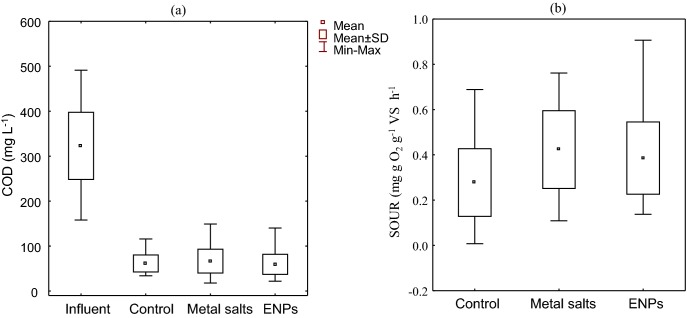
**(a)** Effect of mixed ENPs and metal salts COD removal. **(b)** Influence of treatment on the SOUR of AS microbial community. SOUR values are mean of triplicate determinations.

The median residual ammonia concentration in the three treatment plants was below 1 mg L^−1^ indicating that all pilot plants were operated at optimum and were not negatively influenced by the applied doses of dissolved metals or ENPs. Similarly, COD removal was unaffected (Fig. 1a). The robustness of the system to metal salts or ENPs spiking is consistent with a previous study (Liang, Das and Hu 2010) in which no adverse effect was observed on organic matter removal by heterotrophic bacteria at 0.75 mg L^−1^ of Ag^0^ in AS after 12 h shock load. Further to this, 0.5 mg L^−1^ of Ag^0^ in a simulated wastewater treatment process barely affected the biological removal of COD and NH_3_^−^ and COD (Hou *et al.* 2012). In contrast, the respirometric analysis showed that the median SOUR (mg O_2_ g^−1^ VS h^−1^) of the metal salts and ENPs AS reactors were >1.5 times higher than in the control reactor (Fig. 1b). Although it is unknown at the moment how ENPs or metal salts interacted with wastewater components to enhance SOUR, it is possible that the increased oxygen uptake is due to microorganisms responding to the physiological stress caused by the presence of the ENPs and/or metals (von Moos and Slaveykova 2014).

### Changes in floc size, suspended and volatile solids

A significant difference was observed in the median floc size of the ENP spiked reactor compared to the control (*P* < 0.05) (Fig. S2, Supporting Information). The alteration in the distribution was more pronounced for the larger aggregate size bands with the decrease in the d(0.1) being less than anticipated if the dose was disrupting floc formation. Accordingly, the observed results indicate that the presence of inorganic materials is acting as seed connection points altering the nature of the aggregates and resulting in difference in shape. Confirmation is provided through fractal dimension (df) analysis which indicates a change in df from 771 μm in the control to 568 and 696 μm for the ENP and metal salt spiked reactors, respectively. The structural changes in the aggregates resulted in a decreased settleability as evidenced through a statistically significant (*P* < 0.05) increase in the SVI that changed from 85 ± 27 mL g^−1^ in the control to 152 ± 68 mL g^−1^ and 100 ± 28 mL g^−1^ for the metal and ENP spiked reactors, respectively (Table 1). The MLSS and MLVSS were significantly lower in the ENP and metal spiked AS reactors compared to the control (*P* < 0.05) (Table S2, Supporting Information). The results suggest that ENPs and metal salts have antimicrobial properties which can reduce synthesis of extracellular polymeric substances and therefore floc formation (Liu *et al.* 2007).

### Effects of ENPs on bacterial abundance and survival

**Figure 2.**
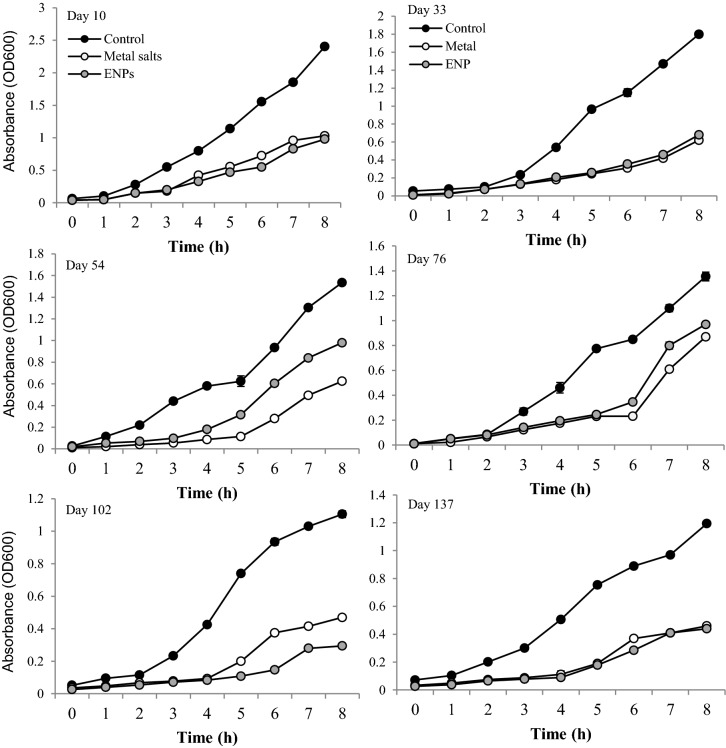
Effect of ENPs and metal salts on the microbial growth kinetics after 8 hours.

The abundance of *E*scherichia *coli*, coliforms and heterotrophs mirror the change in MLSS (see Table S3, Supporting Information) which were significantly decreased (*P* < 0.05) compared to the control. Microbial abundance, however, barely changed overtime with a survival rate of approximately 80% of all cultivable microbes. This was influenced by the continuous-flow design of the pilot plants. Temporal delay in microbial growth was evident from the OD readings of the samples spiked with the ENPs or metal salts compared to the control (Fig. 2). Growth inhibition ranged between 2–85% in ENP and 12–88% in metal salts spiked reactors, although a mean of 58 and 57% inhibition respectively indicates that treatment had similar effect on the microbial growth dynamics (Fig. 2). This finding illustrates that ENP can interact with sludge bacterial community in ways that differ from the bulk metal salts. The practical implication is that ENPs can augment microbial reaction rate and simultaneously inhibit growth and abundance of some microorganisms, e.g. SOUR increased with no observed inhibitory effect on nitrification, whereas a longer lag phase in heterotrophic bacterial growth was observed compared to the control.

### Effects of ENPs on microbial cell structure

**Figure 3.**
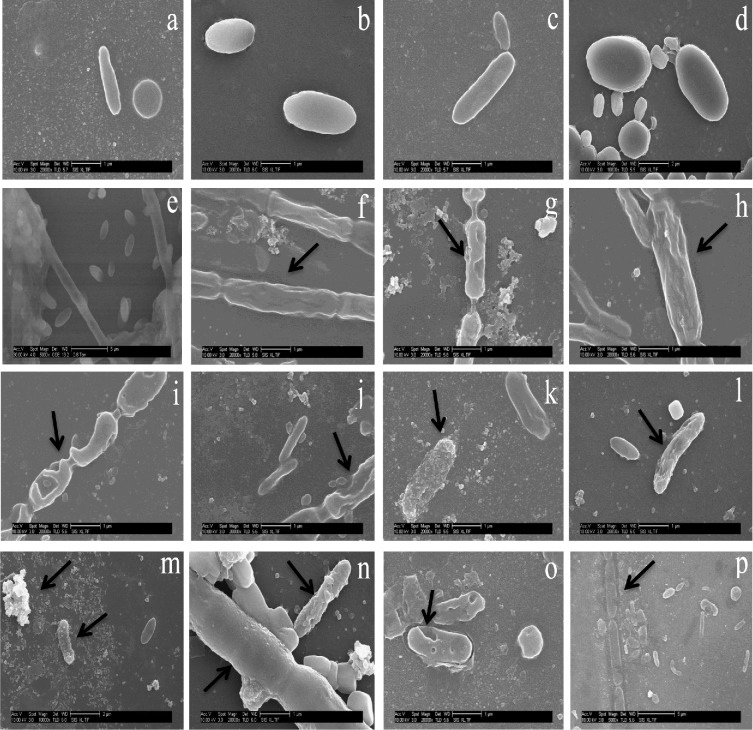
Representative SEM images of ENPs and metal salts effect on AS microbial cells in relation to control after 60 days exposure. (**a–e**) Intact microbial cells in control; (**f, h**) metal salts distorts and shrinks microbial cell; (**g**) perforations of cells by metals salts; (**i, j**) cell wall perforation by ENPs; (**k**) selective adsorption of ENPs to cells; (**l**) selective cell degenerated by ENPs ions or ROS; (**m**) selective adsorption to cell and aggregation of ENPs; (**n**) sheathed and unsheathed cell damage by ENPs; (**o**) cell wall perforations by ENPs; (**p**) ENP dissolves cell wall and sheath.

Representative SEM images of the microbial biomass exposed for 60 days to mixed ENPs and metal salts compared to the control evidenced visible and varying damage to some microbial cells (Fig. 3). Mixed ENPs disrupted bacterial cell integrity by forming pits on the cell wall (panels k, l, m). This suggests that the rate at which the mixed ENPs were repulsed or attracted to the cells differed and can be a result of charges on bacterial cell wall influenced by wastewater components (Sondi and Salopek-Sondi 2004; Eduok *et al.* 2013). Metal salts in contrast caused congealing, shrinking and distortion of the microbial cell wall (panels f, h vs panels i, j). However, it was difficult to determine which ions caused the damage on the microbial cells observed. Images in panels i, j, k, l indicate that the presence of ions influenced ENP reaction. Contact through ion formed in the AS is an important mechanism of ENPs toxicity on bacterial cells which is exacerbated by the pH of the wastewater (Sondi and Salopek-Sondi 2004). For instance, ROS formed by TiO_2_ is enhanced at basic pH values (Kormann, Bahnemann and Hoffmann 2991). pH of the influent wastewater (Fig. S3, Supporting Information) and the HRT of 8 h likely contributed to ROS formation in the AS. Overall, the synergistic or additive effects of ions generated by the low concentration of mixed ENPs and the wastewater components enhanced the biological removal rate of the bulk contaminants. The most plausible explanation for this phenomenon is the Trojan horse effect (Park *et al.* 2010) in which mixed ENPs interacted with wastewater components in part to enhanced microbial activity, and in part caused damage to the microbial cells.

### Effects of ENPs on microbial community structure

Thirteen lipid biomarkers were identified to represent the bacterial PLFA of the AS community (Fig. S4, Supporting Information). Ten biomarkers occurred at less than 100 μg g^−1^ concentration with the exception of 16:0, 18:1ω7t and 16:1ω7c. The results indicate the dominance of *Proteobacteria*, *Bacteriodetes*, *Firmicutes* and *Actinobacteria* (Table S4, Supporting Information). This pattern of dominance in bacterial groups was maintained in all samples indicating that there was no rapid change in the bacterial response to the altered environmental conditions. Biomarker 16:0 occurs in more than one phylogenetic group such as *Proteobacteria* and *Actinobacteria* (Table S4, Supporting Information; Cloete *et al.* 2003; Quezada *et al.* 2006) which accounted for the high concentration and abundance in the sludge samples. The dominance of the Gram negative bacterial biomarker (18:1ω7t; Table S4 and Fig. S4, Supporting Information) indicates enteric source of the bacterial community (Cloete *et al.* 2003). Aerobic bacterial biomarker (16:1ω7c) occurred in higher concentration than other biomarkers and was expected because of the treatment conditions. The ratio of specific PLFAs such as cyclo/monounsaturated precursor (cy17:0/16:1ω7c and cy19:0/18:1ω7c) usually indicate conditional and post-synthetic modification of cell wall lipids by microorganisms under starvation or physiological stress (Frostegard, Tunlid and Bååth 2010). Therefore, shift in proportions of cy17:0 and 16:1ω7c indicates either an altered bacterial cell wall composition or a microbial species composition change as a result of the treatment. However, it was difficult to delineate which of the two outcomes occurred. Again, the change in cyclopropyl fatty acid to *cis*-monoenoic fatty acid indicating modification of microbial cell wall under stress was more pronounced in the metal salts spiked sludge samples. This interpretation should however be taken with caution because of the complex nature of AS. An increase in the ratio of cy17/16:1ω7c can potentially be misinterpreted as stress condition instead of regrowing of the bacterial community (Frostegard, Tunlid and Bååth 2010). Comparison of the PLFA fingerprints between treatments over time indicates a clear temporal shift in bacterial community structure (data not shown). The result implies that the metal salts and ENPs exerted variable effects on the AS bacterial community structure compared with the control.

### Changes in microbial diversity and population dynamics

**Figure 4.**
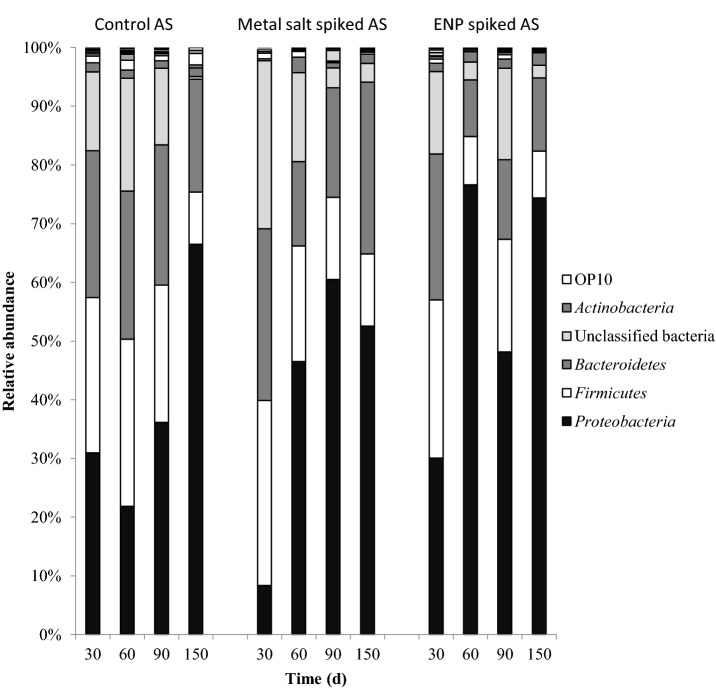
Dominant phyla of AS bacterial community based on 454 pyrosequencing with phylogenetic group above 5% abundance.

To further understand the changes in the AS bacterial community structure and diversity as a result of exposure to the chronic low doses of ENPs and metal salts, 454 pyrosequencing was carried out. Bacterial distribution and diversity at the phylum level for each treatment at different times are summarized in Fig. 4. A total of 17 022 sequences representing 25 classifiable phyla were obtained. A total of 7468, 4130 and 5424 sequences were obtained for the control, metal salts and ENPs spiked sludges, respectively. Most predominant phylotypes were members of the *Proteobacteria*, *Firmicutes*, *Bacteriodetes*, unclassified Bacteria and *Actinobacteria* (Table S5, Supporting Information).

The dominant bacterial phyla in ENPs and metal salts spiked sludges exhibited a clear distribution pattern with temporal changes in diversity and relative abundance (Fig. 4). The change in bacterial community response indicates species-sensitive and species-tolerant to ENPs. For instance, the relative abundance of *Bacteriodetes* in control was stable compared to ENP and metal salts spiked ASs with abundance of the clinically important *Bacteriodes* and *Parabacteroides* usually found in human faeces (Garrity, 2010). *Actinobacteria* was relatively stable in the control and ENP spiked AS. In contrast, the *Firmicutes* decreased overtime in the three AS reactors. Members belonging to *Clostridia* and *Bacilli* were most abundant and both were sensitive to metal salts or ENPs. Also, the relative abundance of unclassified bacteria varied largely probably because most microorganisms in AS are yet to be fully identified (Kragelund *et al.* 2008).

The relative abundance of the dominant groups followed a similar pattern regardless of treatment. Members of the family *Comamonadaceae* (*Burkholderiales*) were identified as the most dominant and resilient bacterial species with *Acidovorax*, *Rhodoferax*, *Comamonas*, *Curvibacter*, *Giesbergia*, *Hydrogenophaga*, *Ottowia* and *Simplicispira* as prominent genera in ENP spiked reactors. Compared with control and metal salts spiked reactors, *Acidovorax* was about two times higher suggesting that the ENP mixture can positively influence the metabolic activities and growth of specific organism. In contrast, ammonia-oxidizing bacteria (AOB) belonging to *Betaproteobacteria* and *Gammaproteobacteria* were not recovered indicating that they were susceptible to ENPs and metal salts. Specifically the non-recovery of the common ammonia- and nitrite-oxidizing bacteria (NOB) such as *Nitrosomonas*, *Nitrobacter* or *Nitrospira* (Table S5, Supporting Information) suggests that ENPs can selectively inhibit microorganisms in AS. Nevertheless, ammonia and nitrite were oxidized which suggest that other nano-tolerant microbial species were involved as explained hereinafter.

**Figure 5.**
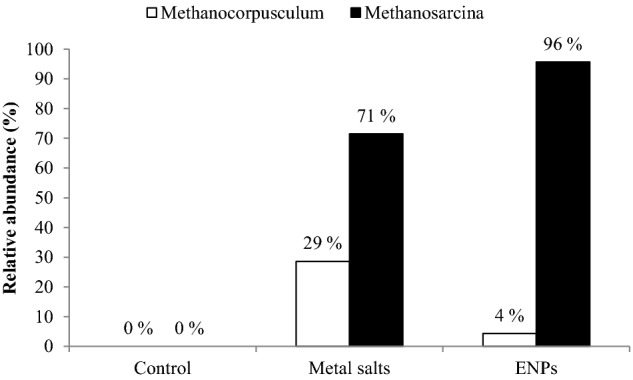
Relative abundance of the *Methanosarcina and Methanocorpusculum* in AS reactors.

The relative abundance of the two identified archaeal members *Methanocorpusculum Methanosarcina* was significantly different between treatments (Fig. 5). Specifically the abundance of *Methanosarcina* was 1.3 times higher in the ENP spiked reactor compared to the metal salt spiked reactors. Further to this, *Methanocorpusculum* was 6.7 times higher in the metal salt spiked than in ENP spiked reactor. Both organisms were undetected in the control (Fig. 5) and the reason for this is unclear at the moment. Contrary to previous and commonly held notion that the *Archaea* were obligate anaerobes, available evidence, however, has shifted the weight of argument in favour of ammonia-oxidizing *Archaea* in AS (Park *et al.* 2006; Prosser and Nicol 2008). Thus, it is reasonable to assume that the spiked concentrations of metal salts and ENPs altered the AS physicochemical composition and created conditions that enhanced the growth of *Methanocorpusculum* and *Methanosarcina*, respectively.

In the absence of the known AOB and NOB belonging to the *Betaproteobacteria* and *Gammaproteobacteria* in the metal salts and ENPs spiked reactors as previously mentioned, it is plausible that the nano-tolerant members of *Burkholderiales, Chloroflexi* and the two archaeal genera with oxygen-dependent respiration were involved in oxidizing ammonia, and removal of organic carbon in the effluent.

## CONCLUSION

The production of biosolids was not reduced by the ENPs or metal salts doses of 0.01 mg L^−1^ Ag^0^, 0.08 mg L^−1^ TiO_2_ and 0.12 mg L^−1^ ZnO. However, the sorption to the biosolids and the microbial community response to the ENPs differed from the bulk metal salts. For instance, ENPs stimulated certain microbes activity such as SOUR but in the meantime inhibited specific microbes, such as *Nitrosomonas* and *Nitrospira*. *Acidovorax, Rhodoferax, Comamonas* and *Methanosarcina* were all tolerant to the ENPs dosing and are likely playing a key role in the nitrification and the contaminant removal from the sludge. While some microbes were tolerant to the ENPs, it was physiologically stressful as evidenced by a shift in the bacterial community structure. This study also showed that ENPs can simultaneously stimulate and inhibit microbial-mediated processes and/or disrupt bacterial cells. In addition, wastewater components can increase or reduce the ENPs concentration available for a time-dependent Trojan horse-like effect on AS microbial community. Thus, ENPs exerted a discontinuous biological effect and the potential hazard on ecologically important microbes can increase or decrease with prevailing condition and reactions in the AS reactor. Overall, the chronic exposure of AS microorganisms to ENP resulted in community tolerance evidenced by species composition shifts and acclimation of individual nano-tolerant organisms.

## Supplementary Material

Supplementary data are available at FEMSEC online.
