# Supplementary material for: Insights into the effect of mixed engineered nanoparticles on activated sludge performance
Source: FEMS Microbiol Ecol. 2015 Aug 4;91(7):fiv082. doi: 10.1093/femsec/fiv082 (PMC4629872; doi:10.1093/femsec/fiv082)
Supplement: Supplementary data are available at FEMSEC online. [file Supporting_Information.docx]

**Supporting information**

**Insights into the effect of mixed engineered nanoparticles on activated sludge performance**

Samuel Eduok, Callum Hendry, Robert Ferguson, Ben Martin, Raffaella Villa, Bruce Jefferson, Frédéric Coulon*

**Number of Figures:** 8

**Number of Tables**: 5

**Figure S1**. Flow schematic of the pilot-scale plant operation with the spiked and targeted ENPs and metal salt concentration in the digestate

**Figure S2:** Particle size distribution of biomass flocs in activated sludge

**Figure S3:** (a) pH and (b) conductivity for influent and effluents of each treatment

**Figure S4:** PLFA concentrations in AS microbial biomass exposed to chronic low concentrations of ENPs and metal salts.

**Figure S5:** Relative abundance of *Proteobacteria* in AS indicating the dominance of members of the *Betaproteobacteria* and resilience of the Alphaproteobacterial subgroups to the effect of treatment compared with the control. Group above 1 % abundance in the AS are shown.

**Figure S6**: Relative abundance of members of the *Comamonadaceae* (β-*Proteobacteria*) showing *Acidovorax* and *Rhodoferax* with the highest percentage recovery in the subgroup and bacterial community. Species with at least 1 % abundance in the AS are indicated. * = Unclassified

**Figure S7**: Effect of treatment on the relative abundance of the phylum *Firmicutes* in activated sludge. Members of Class Clostridia were most abundant among the *Firmicutes*.

**Figure S8**: Effect of treatment on the relative abundance of the *Bacteriodetes* in AS.

The ENPs concentrations in the activated sludge were determined as part of a broad treatment design to produce a digestate sludge cake containing titanium (2000 mg kg⁻ˡ), Zinc oxide (2800 mg kg⁻ˡ) and silver oxide (250 mg kg⁻ˡ), the maximum level permitted in sewage sludge solids applied to agricultural land as defined by the US EPA. 80 – 90% of the nanoparticles in wastewater matrices associate with solids in the sewer network or during primary sedimentation. The remaining 10-20% make it into secondary treatment. To ensure the correct or slightly exceeded target levels in the final solid products, we dosed concentrations into the activated sludge at 28% of the daily amount added to the anaerobic digesters (which were fed from solids derived from primary settling). The levels of ENPs dosed into the pilot plant are shown in Table S1

**Table S1:** Concentration of ENPs and metal salts spiked into the reactors

| Engineered nanoparticle | Activated sludge |
| --- | --- |
| Silver oxide (mg kg⁻ˡ) | 11.11 |
| Titanium dioxide (mg kg⁻ˡ) | 88.88 |
| Zinc oxide (mg kg⁻ˡ) | 124.44 |

**Table S2:** Effect of ENP and metal salt ions on biosolids and bulk removal of nitrogenous compounds in pilot plant activated sludge over 315 days of treatment

| Parameter | Influent | Control | Metal salts spiked | ENP spiked |
| --- | --- | --- | --- | --- |
| N₂ (mg L⁻ˡ) | 26 ± 6 | 35.6 ± 10.2 | 37.5 ± 12 | 37.8 ± 9.3 |
| NO₂ (mg L⁻ˡ) | 0.06 ± 0.05 | 0.62 ± 0.14 | 0.79 ± 0.36 | 0.59 ± 0.18 |
| NO₃ (mg L⁻ˡ) | 0.67 ± 0.2 | 34.2 ± 4 | 30.3 ± 6 | 33 ± 5 |
| MLSS (mg L⁻ˡ) | na | 1970 ± 399.7 | 1490 ± 200 | 1630 ± 198.6 |
| MLVSS (mg L⁻ˡ) | na | 1550 ± 300 | 1140 ± 199.8 | 1200 ± 199.7 |

**Table S3:** ENPs and metal salts effect on culturable microbial groups in activated sludge over 180 days of treatment

| Treatment | Microbial counts (Log₁₀ CFU mL⁻ˡ) | | |
| --- | --- | --- | --- |
|  | Heterotrophs | Coliforms | *E.coli* |
| Influent wastewater | 8.8 ± 0.4 (na) | 7.8 ± 0.3 (na) | 7.9 ± 0.05 (na) |
| Control | 7.7 ± 0.4 (na) | 5.9 ± 0.07 (na) | 5.8 ± 0.07 (na) |
| ENP spiked | 5.9 ± 0.07  (77 ± 4) | 4.8 ± 0.1  (81 ± 1.5 ) | 4.7 ± 0.2  (81 ± 5 ) |
| Metal salt spiked | 5.8 ± 0.04  (76 ± 4) | 5.0 ± 0.4  (86 ± 8) | 4.6 ± 09  (79 ± 1.4) |

Values in parenthesis represent the percentage survival of culturable microbial groups in activated sludge spiked with ENPs and metal salts, AS = activated sludge, na = not applicable

**Table S4**. Phospholipid fatty acid markers of selected microbial groups in the sludge

| **Lipid biomarker** | **Phylogenetic**  **affiliation** | **Type** | **Related microbial group/genus/species** | **References** |
| --- | --- | --- | --- | --- |
| Gram positive bacteria |  |  |  |  |
| 14:0, i15:0, 16:0, i16:0, i17:0, 16:1ω5, 18:1ω7, 19:0cy, 16:1ω7 | *Firmicutes, ProteobacteriaBacteriodetes* | Facultative anaerobe | *Streptococcus, Pediococcus, Enterococcus, Staphylococcus, Bacillus, Pectinatus* | Quezada et al., 2006; Oravecz et al., 2004; Cloete et al., 2003 |
| i15:0, i16:0, i17:0, 16:1ω5, 18:1ω7t | *ProteobacteriaActinobacteria* | Aerobe, Anaerobe, Aerotolerant | Sulfate-reducing*, Lactobacillus, Desulfobacter, Desulfovibrio desulfuricans, Streptomyces, Bacillus licheniformis,* | Lai and Shao, 2008; Quezada et al., 2006; Oravecz et al., 2004, Cloete et al., 2003; Richie et al., 2000 |
| i16:0, 10Me18:0 | *Actinobacteria* | Aerobe | *Corynebacterium, Gordonia, Rhodococcus, Norcardia, Arthrobacter, Tsukamurella, Micrococcus,Mycobacterium* | Lai and Shao, 2008; Quezada et al., 2006; Oravecz et al., 2004; Richie et al., 2000; Cloete et al., 2003 |
| Gram negative bacteria |  |  |  |  |
| 14:0, 17:0cy, 19:0cy | *Proteobacteria* | Facultative anaerobe | *Enterobacter, Vibrio* | Oravecz et al., 2004 |
| 16:0 | *Proteobacteria* | Aerobe | *Pseudomonas* | Haack et al., 1988 |
| ai15:0, 16:1ω5, 17:0cy, 19:0cy | *Proteobacteria* | Anaerobe | *Xanthomonas, Pseudomonas, Arthrobacter* | Oravecz et al., 2004; Haack et al., 1988 |
| 16:1ω5 | *Bacteriodetes, Firmicutes* | Aerobe | *Cytophaga, Flavobacterium* | Frostegard et al., 1993; Kelly et al., 1999; Oravecz et al., 2004 |
| 16:1ω5, 16:0 | Fungi/Bacteria |  | *Arbuscular mycorrhiza* | Quezada et al., 2006; Oravecz et al., 2004; Richie et al., 2000; Olsson et al., 1995,Olsson, 1999; Kelly et al., 1999 |

**Table S5**. Summary of the most dominant phylogenetic groups of bacteria in activated sludge based on 454 Pyrosequencing

| Cluster | Phylogenetic affiliation | Order/Family/Genus | *Genus/Description* | % Match | Accession No. |
| --- | --- | --- | --- | --- | --- |
| 112 | Proteobacteria | Betaproteobacteria/Rhodocyclales/Rhodocyclaceae/*Quatrionicoccus* | *Qautrionicoccus australiensis* strain Ben 117 | 99 | NR029035 |
| 306 |  | Betaproteobacteria/Burkholderiales/Comamonadaceae/*Comamonas* | *Comamonas* sp LYS1-1 | 97 | KC211010 |
| 359 |  | Alphaproteobacteria | *Sphingopyxis* sp MC1 | 88 | JN940802 |
| 395 |  | Betaproteobacteria/Burkholderiales/Incertae sedis 5/*Methylibium* | *Methylibium* sp BAC116 | 99 | EU130971 |
| 485 |  | Alphaproteobacteria/Rhodobacterales/Rhodobacteraceae/*Rhodobacter* | *Rhodobacter* sp TUT3732 | 93 | AB251408 |
| 502 |  | Betaproteobacteria/Burkholderiales | *Acidovorax* sp BSB421 | 94 | Y18617 |
| 542 |  | Betaproteobacteria/Burkholderiales/Comamonadaceae/*Rhodoferax* | *Rhodoferax* sp B3 | 98 | DQ268771 |
| 564 |  | Alphaproteobacteria/Rhodobacterales/Rhodobacteraceae/*Rhodobaca* | *Rhodobacter* sp TCRI 3 | 94 | AB017796 |
| 649 |  | Alphaproteobacteria/Rhizobiales | *Oligotropha carboxidovorans* | 90 | AB099659 |
| 727 |  | Alphaproteobacteria/Sphingomonadales/Sphingomonadaceae/*Sphingopyxis* | *Sphingopyxis witflariensis* strain W-50 | 99 | NR028010 |
| 810 |  | Deltaproteobacteria | *Dongia mobilis* strain LM22 | 83 | FJ455532 |
| 983 |  | Alphaproteobacteria/Sphingomonadales/Sphingomonadaceae/*Novosphingobium* | *Novosphingobium hassiacum* strain W-51 | 99 | NR028962 |
| 1522 |  | Gammaproteobacteria/Xanthomonadales/Xanthomonadaceae/Xylella | *Pseudoxanthomonas* sp E16 | 93 | AY488509 |
| 1579 |  | Alphaproteobacteria/Rhizobiales/Hyphomicrobiaceae/*Hyphomicrobium* | *Hyphomicrobium* sp M3 | 97 | AF098790 |
| 1696 |  | Gammaproteobacteria/Pseudomonadales/Moraxellaceae/*Acinetobacter* | *Acinetobacter iwoffii* strain JUN-5 | 97 | KF228924 |
| 1706 |  | Gammaproteobacteria/Pseudomonadales/Pseudomonadaceae/*Flavimonas* | *Pseudomonas* sp HY-14 | 98 | EU620679.2 |
| 1773 |  | Betaproteobacteria/Burkholderiales/Comamonadaceae | *Simplicispira metamorpha* strain DSM 1837 | 94 | NR044941 |
| 1997 |  | Betaproteobacteria/Burkholderiales/Comamonadaceae/*Curvibacter* | *Curvibacter* sp W2 09-301r | 95 | JX458451 |
| 2143 |  | Betaproteobacteria | *Rhodoferax ferrireducens* | 90 | AF435948 |
| 2271 |  | Betaproteobacteria/Burkholderiales/Comamonadaceae/Giesbergeria | *Acidovorax ebreus* TPSY strain TPSY | 95 | NR074591 |
| 2410 |  | Betaproteobacteria/Burkholderiales/Comamonadaceae/*Acidovorax* | *Acidovorax defluvii* strain BSB411 | 93 | NR026506 |
| 2490 |  | Alphaproteobacteria/Rhodobacterales/Rhodobacteraceae/*Paracoccus* | *Paracoccus* sp BBTR62 | 98 | DQ337586 |
| 2592 |  | Alphaproteobacteria/Sphingomonadales/Sphingomonadaceae/*Sphingosinicella* | *Spingosinicella microcystinivorans* | 95 | AB219940 |
| 2608 |  | Alphaproteobacteria/Rhodobacterales/Rhodobacteraceae/*Pseudorhodobacter* | *Rhodobacter* sp TUT3734 | 96 | AB251410 |
| 2739 |  | Gammaproteobacteria/Xanthomonadales/Xanthomonadaceae/*Dokdonella* | *Dokdonella* sp LM 2-5 | 93 | FJ455531 |

**Table S5** continued.

| Cluster | Phylogenetic affiliation | Order/Family/Genus | Genus/Description | % Match | Accession No. |
| --- | --- | --- | --- | --- | --- |
| 2823 | Proteobacteria | Alphaproteobacteria/Rhizobiales/Bradyrhizobiaceae/Bosea | Bosea thiooxidans strain E14 | 98 | AY488508 |
| 3277 |  | Alphaproteobacteria/Rhodobacterales/Rhodobacteraceae | Paracoccus aminophilus JCM7686 | 95 | CP006650 |
| 3310 |  | Betaproteobacteria/Burkholderiales/Incertae sedis 5/Aquabacterium | Aquabacterium commune strain B8 | 97 | NR024875 |
| 3597 |  | Gammaproteobacteria/Xanthomonadales/Xanthomonadaceae/Thermomonas | Xanthomonas axonopodis | 99 | AB101447 |
| 29 | Firmicutes | Clostridia/Clostridiales/Eubacteriaceae/Eubacterium | Eubacterium sp E-1 | 99 | AB470313 |
| 269 |  | Bacilli/Lactobacillales/Lactobacillaceae/Lactobacillus | Lactobaccillus vaginalis strain DoxG3 | 97 | GQ422709 |
| 380 |  | Clostridia/Clostridiales/Incertae Sedis XI/Sedimentibacter | Sedimentibacter hongkongensis strain K1 | 88 | AY571338 |
| 391 |  | Clostridia | Clostridium sp strain Z6 | 83 | AY949859 |
| 475 |  | Clostridia/Clostridiales/Syntrophomonadaceae | Synthrophomonas curvata strain GB8-1 | 89 | NR025752 |
| 530 |  | Clostridia/Clostridiales/Ruminococcaceae | Ruminococcus sp 15975 | 84 | AJ308104 |
| 776 |  | Clostridia/Clostridiales/Clostridiaceae/Clostridiaceae 4/Caminicella | Proteocatello sphenisci strain PPP2 | 94 | NR041885 |
| 844 |  | Bacilli/Lactobacillales/Enterococcaceae/Enterococcus | Enterococcus sp R-25205 | 94 | AM084029 |
| 845 |  | Clostridia/Clostridiales | Fecalibacterium prausnitzii strain HTF-E | 82 | HQ457029 |
| 899 |  | Bacilli/Bacillales/Bacillaceae/Bacillaceae 1/Bacillus/Bacillus h | Bacillus sp RCT10 | 90 | FJ755951 |
| 1147 |  | Clostridia/Clostridiales/Incertae Sedis XI/Sedimentibacter | Sedimentibacter sp MO-SED | 90 | AB598275 |
| 1276 |  | Clostridia/Clostridiales | Clostridiales bacterium JN18 A24 | 91 | DQ168655 |
| 1382 |  | Clostridia/Clostridiales/Clostridiaceae/Clostridiaceae 1/Clostridium | Clostridium sp | 92 | X95274 |
| 1655 |  | Clostridia/Clostridiales/Ruminococcaceae/Ethanoligenens | Ethanoligens harbinense strain CGMCC1152 | 90 | AY833426 |
| 1921 |  | Clostridia/Clostridiales/Incertae Sedis XV/Aminobacterium | Aminobacterium mobile strain ILE-3 | 99 | NR024925 |
| 2477 |  | Bacilli/Lactobacillales/Enterococcaceae/Enterococcus | Enterococcus devriesei strain LMG 13603 | 97 | DQ010644 |
| 2513 |  | Clostridia/Clostridiales/Clostridiaceae/Clostridiaceae 1/Clostridium | Clostridium thiosulforeducens | 95 | AF317650 |
| 2976 |  | Clostridia/Clostridiales/Ruminococcaceae | Oscillibacter sp G2 | 91 | HM626173 |
| 3227 |  | Clostridia/Clostridiales/Peptostreptococcaceae/Peptostreptococcaceae Incertae Sedis | Clostridium metallolevans strain SN1 | 94 | EU887815 |
| 3305 |  | Clostridia/Clostridiales/Lachnospiraceae | Clostridium glycyrrhizinilyticum | 92 | AB233029 |
| 3572 |  | Clostridia/Clostridiales/Veillonellaceae/Selenomonas | Selenomonas ruminantium | 91 | AB198442 |

| Cluster | Phylogenetic affiliation | Order/Family/Genus | Genus/Description | % Match | Accession No. |
| --- | --- | --- | --- | --- | --- |
| 43 | Bacteroidetes | Sphingobacteria/Sphingobacteriales/Flexibacteraceae/Niastella | Niastella sp Gsoil 221 | 88 | GQ339899 |
| 44 |  | Sphingobacteria/Sphingobacteriales | Myroides sp XJ193 | 78 | GQ381279 |
| 62 |  | Bacteroidales/Porphyromonadaceae/Parabacteroides | Parabacteroides distasonis ATCC 8503 | 89 | CP000140 |
| 362 |  | Bacteroidales/Rikenellaceae/Petrimonas | Proteiniphilum acetatigenes strain TB 107 | 94 | NR043154 |
| 662 |  | Bacteroidales | Cytophaga sp | 81 | X85210 |
| 1252 |  | Bacteroidales | Parabacteroides distasonis strain JCM 5825 | 81 | EU136681 |
| 1344 |  | Flavobacteria/Flavobacteriales/Flavobacteriaceae/Flavobacterium | Flavobacterium columnare strain E8 | 92 | AY488506 |
| 2155 |  | Flavobacteria/Flavobacteriales/Flavobacteriaceae/Flavobacterium | Flavobacterium swingsii | 97 | AM934651 |
| 2259 |  | Bacteroidales/Porphyromonadaceae/Paludibacter | Paludibacter propionicigens WB4 | 86 | CP002345 |
| 2464 |  | Bacteroidales/Porphyromonadaceae/Proteiniphilum | Proteiniphilum acetatigenes strain TB 107 | 84 | NR043154 |
| 3214 |  | Bacteroidales/Rikenellaceae/Marinilabilia | Marinilabilia sp AK2 | 87 | FN994992 |
| 3270 |  | Bacteroidetes | Bacteroides sp strain Z4 | 90 | AY949860 |
| 3593 |  | Bacteroidales/Porphyromonadaceae/Parabacteroides | Parabacteroides johnsonii DSM 18315 strain JCM 13406 | 86 | NR041464 |
| 281 | Actinobacteria | Actinobacteridae/Actinomycetales/Micrococcineae/Microbacteriaceae/Leucobacter | Leucobacter komagatae | 95 | DQ083486 |
| 565 |  | Actinomycetales/Micrococcineae/Microbacteriaceae | Microbacterium sp RI 2 | 94 | AJ876685 |
| 1619 |  | Actinomycetales/Propionibacterineae/Propionibacteriaceae/Propionibacterium | Propionibacteriumsp SV442 | 94 | AB264627 |
| 3130 |  | Actinomycetales/Corynebacterineae/Mycobacteriaceae/Mycobacterium | Mycobacterium aichiense strain JS618 | 97 | AF498656 |
| 3625 |  | Actinomycetales/Micrococcineae/Microbacteriaceae | Microbacterium thalassium | 98 | AM943052 |
| 2484 | Fusobacteria | Fusobacteriales/Fusobacteriaceae/Fusobacterium | Fusobacterium sp SRBBR5 | 99 | HM215007 |
| 883 | Chloroflexi | Chloroflexi/Anaerolineae | Bacterium JN18 A7 F* | 96 | DQ168648 |

sp = species
